# Towards multilingualism in global health

**DOI:** 10.1186/s12992-025-01107-6

**Published:** 2025-04-03

**Authors:** Ralph Hurley O’Dwyer, Rebecca C. Stout, Émilie S. Koum Besson, Amaya L. Bustinduy, Máire A. Connolly

**Affiliations:** 1 Department of Public Health, HSE Dublin and Midlands, Dr Steevens’ Hospital, Dublin, Ireland; 2 School of Health Sciences, University of Galway, Galway, Ireland; 3 Department of Clinical Research, London School of Hygiene and Tropical Medicine, London, UK; 4 Department of Infectious Diseases, Imperial College London, London, UK; 5 Independent Researcher, Cotonou, Benin; 6 Insight SFI Centre for Data Analytics, Galway, Ireland

## Abstract

A forgotten aspect of the decolonizing global health movement is the impact of monolingualism on the practice of medicine and global health. Thousands of languages are spoken worldwide yet remarkably few are used in these fields. English, in particular, plays an extraordinarily dominant role. The status of English as the global medical lingua franca perpetuates inequities in research, medical education and healthcare delivery, disproportionately affecting many low-and middle-income countries (LMICs). This linguistic hegemony creates barriers to accessing health information for minoritized populations and discriminates against researchers from non-native English-speaking backgrounds. Even the speakers of major world languages such as Arabic and Hindi are marginalized, with little research published in these languages and medical education generally unavailable in them. This inequality affects patients’ ability to receive care and access information in their own languages and contributes to mistrust and exclusion. This is particularly the case in formerly colonized countries where exploitative medical practices remain a painful legacy. A paradigm shift is urgently needed in the global health field to address these inequities. We propose solutions include expanding foreign language education, supporting minoritized languages in health promotion, and mandating the dissemination of research output in the languages of the studied populations. Ultimately, the languages we choose to use as global health practitioners shape power dynamics, determine whose voices are heard, and impact the effectiveness of our actions. Without urgent and systemic change, the dominance of a few languages, particularly English, risks perpetuating inequities and excluding those most in need of inclusion.

## Introduction

In 1921, the philosopher Ludwig Wittgenstein noted: “The limits of my language mean the limits of my world”. Whereas this statement concerns a debate beyond the premise of this article, Wittgenstein’s words nonetheless are profoundly relevant to the importance of multilingualism in global health. This article describes the challenges posed by the dominance of English in medicine globally and the importance of language in decolonizing global health. The dominance of English in the medical field favors native speakers and poses obstacles to the publication of medical research in many regions of the world. Furthermore, it limits access to medical information in many low-and middle-income-countries (LMICs) and can negatively impact health care delivery in development and humanitarian settings, exacerbating pre-existing colonial structures. We propose potential solutions to this current linguistic hegemony and illustrate the advantages of encouraging multilingualism in the global health field.

## Defining multilingualism

Multilingualism can be defined as the use of multiple languages by an individual speaker or a group of speakers, not necessarily equally. Both historically and currently, there have been more multilinguals than monolinguals, making multilingualism the societal default [1]. To this day, in large regions of the world, particularly in many low- and middle-income countries, it is common for individuals to speak and use multiple languages on a daily basis [1].

## English as a medical lingua franca – a barrier to medical research in low-and middle-income countries

For the last century, English has been the *lingua franca* of medicine and scientific research [2]. Whereas in the 1800s, doctors were expected to have a knowledge of other major European languages to keep up to date with medical literature, the linguistic hegemony of English in the medical sphere has become increasingly established in recent decades [2]. This linguistic monopoly is unusual given approximately only 5% of the world’s population speak English as their first language, with less than 20% being able to speak it as a second language [3]. In addition, despite the nascent decolonizing global health movement, it has been noted that most global health literature is also published only in English [4]. This dominance is such that even researchers with other major global languages such as French as their native language, have noted the barriers they face in medical research, describing linguistic isolation and discrimination in their field [5].

Kamadjeu argues that English has evolved to be the global lingua franca of scientific research through Darwinian forces and that the pragmatism of having one such global lingual franca outweighs the negative effects of this linguistic monopoly [6]. Yet the potential harms of such a monopoly are multiple. The widespread acceptance of English as a lingua franca in medicine results in a power imbalance between native and non-native English speakers, given the time and economic investment necessary for non-native speakers to be able to write a journal article and present at a conference in the language. For example, a researcher in Dakar with Wolof as their native language and French as their second language faces significant additional challenges in their career compared to their British or even Nigerian colleagues. Moreover, these inequities exist not only individually but also at national and international levels. The use of English as a lingua franca significantly privileges countries in the Anglosphere who are not obliged to invest substantial state resources into learning it. Furthermore, despite its role as a global lingua franca, English cannot escape its role as the national language of various states, resulting in possible political biases and tensions, given the UK’s colonial past and the current geopolitical dominance of the USA.

Furthermore, the overwhelming majority of medical journals in Africa are in English with most research conducted in Africa also published in English, a language spoken natively by very few in the continent [7]. Although many African researchers speak English, many of the communities in which research is undertaken do not, meaning they may never access the knowledge to which they contribute. Financial barriers are an important consideration too given that a majority of grant applications are in English [7].

## Linguistic hegemony as a barrier to equitable access to health information globally

The global dominance of a small number of languages obstructs equitable access to health information. Minoritized populations who do not have access to accurate, timely information in their own languages may be more likely to resort to inaccurate information, otherwise termed “fake news” [8]. This may have important effects on other criteria such as the low vaccination levels documented within certain minority ethnolinguistic populations in Europe [8].

## The impact of linguistic hegemony on medical education and practice globally

The paucity of languages used in medical education globally has numerous negative effects though is rarely discussed. There are over 6000 languages spoken worldwide. A global database of medical colleges showed medical education is delivered in approximately only 50 of them, with most medical resources published in significantly fewer [9]. Even some of the most widely spoken languages, such as Arabic and Hindi, are rarely used in medical education [10, 11]. Arabic, while spoken by approximately 350 million people in all its varieties, and an official language in 26 countries, is only used as the principal language of medical education and medical communication in one country – Syria [11]. This peculiar situation has multiple knock-on effects, which may affect the ability of Arabic speaking patients and healthcare practitioners to access health information and, in some cases, even healthcare in their own language [12].

Medical education in many post-colonial nations continues to be delivered in English or French, even when neither instructors nor students are native speakers of the relevant language. For example, in Algeria, medical courses are taught in French, despite the fact that both students and the majority of faculty speak Algerian Arabic as their first language and communicate with patients in Algerian Arabic [12]. This phenomenon—where medicine is “lost in translation” even between speakers of a shared native language—can create serious challenges in patient care. Whereas barriers exist to effective communication between healthcare practitioners and patients in languages such as English due to medical terminology jargon [13], this gap becomes greater when patients must speak another language to understand the vocabulary of their healthcare provider [14]. One study from Saudi Arabia showed that many Arab medical students felt unconfident in communicating with patients in their native language [15]. The lack of providing medical education in languages such as Arabic is one of the factors in the lack of appropriate medical resources in the language, itself the foremost obstacle to using it clinically as per another study [16].

## Potential solutions

Numerous solutions exist to help promote multilingualism in global health and address the inequities created by the current dominance of English.

Expanding foreign language education in medical and global health training is a crucial step towards mitigating the linguistic barriers that often undermine international humanitarian and development work. It also plays a key role in fostering a linguistically diverse workforce capable of providing language-concordant care [17]. One notable example is the Voices in Global Health Program at Duke University, where students discuss global health subjects in either French, Spanish, Arabic, Hindi or Mandarin [18]. It is worth noting however, that many of these languages are ones of former colonial powers not of the historically colonized. A truly decolonized global health curriculum should also strive to incorporate the Indigenous languages of the Global South.

Expanding the provision of bilingual medical education, such as the Medical Spanish pathway at Geisel School of Medicine (Dartmouth College), or programmes such as the bilingual French-German program at the University of Fribourg, is also important [19, 20]. Most importantly, medical curricula must reflect the linguistic realities of the populations being served. One promising initiative is the introduction of bilingual Hindi-English medical education in the state of Madhya Pradesh in India [21]. Such programmes may help address the linguistic disconnect that can emerge when doctors are trained in colonial languages, not spoken by the population at large. Beyond language instruction, ensuring that medical school cohorts reflect the linguistic and cultural diversity of a given country’s population is paramount to facilitating a workforce better-equipped for language-concordant care.

The advent of online language education further enables healthcare practitioners to learn relevant languages remotely, while also supporting teachers in LMICs. Requiring healthcare workers to demonstrate proficiency in the primary language spoken by the populations they serve may enhance the effectiveness of development and humanitarian programs. While we advocate for the provision of foreign-language training in medical schools, we also recognize that language education begins at an early age. Therefore, while introducing such training at the medical school level is laudable, systematic change across the general education system is necessary to achieve widespread advanced proficiency in multiple languages, particularly in Anglophone countries and former colonial powers. This requires a radical paradigm shift in the cultural understanding of language and its power in these nations.

Promoting foreign language education may also encourage the consumption and dissemination of research material in languages other than English. A key strategy is to mandate the inclusion of multilingual abstracts, ensuring that at least the abstract of a study is available in the main language of the population amongst which that research was undertaken [3]. While journals such as the Pan American Journal of Public Health, Global Health Promotion and the Bulletin of the World Health Organization already publish abstracts - and in some cases, entire articles in multiple languages, this remains the exception rather than the norm. Funding bodies should also be incentivized to publish research in multiple languages.

Furthermore, it is vital that the speakers of indigenous and minoritized languages are not excluded from participating in research trials based on an inability to speak the dominant language in their area. Flood and Rohloff describe cases of this phenomenon in Guatemala where Spanish-language competence has been an inclusion criterion in certain research trials, excluding Mayan-speaking monolinguals who historically have suffered disproportionately poor health compared to their Spanish-speaking compatriots [22].

Supporting the provision of suitably qualified, regulated and fairly compensated medical interpreters is another pivotal step towards ensuring linguistically equitable healthcare. Countries differ widely in the regulation of medical interpreting with some, such as Ireland, lacking formal regulation or training requirements [23]. Best practice includes programs such as the Medical Interpreter Services Program at Massachusetts General Hospital which employs full-time, qualified medical interpreters on-site [24]. Governments must implement robust regulation to ensure high-quality, accessible medical interpreting services, particularly in linguistically diverse populations.

In many colonized countries, a history of exploitative medical research practices has led to mistrust amongst local communities. Supporting the use of native languages to discuss and practice medicine could be said to combat what the Kenyan author Ngũgĩ wa Thiong’o described as “mental colonization” and “colonial alienation”, which occurs when individuals must use colonial languages to discuss scientific topics such as medicine [25]. Promoting the use of native, indigenous, non-colonial languages in research and health promotion is thus particularly important. Financial support should be provided to support the publication of health information in these languages. One example of this is the work of “Decolonise Science,” which aims to translate 180 papers authored by African researchers into six languages rarely used in the scientific and medical field currently: isiZulu, Northern Sotho, Yoruba, Hausa, Amharic and Luganda. As its founders describe, this not only broadens the transmission of science to people who cannot access literature in English or French but can also “integrate the facts and methods of science into cultures that have been denied [them] in the past” [26].

Lastly, leveraging the power of artificial intelligence (AI) holds great promise in fostering a more linguistically equitable global health landscape. AI can offer an alternative to costly language editing services for non-native English speakers seeking to publish research in English [27]. Moreover, it can facilitate the translation of health information into minoritized languages, improving accessibility for underserved populations. While AI cannot yet replace human interpreters, it represents a valuable adjunct in making healthcare more linguistically inclusive.

## Conclusion

The limits of the languages we use as global health practitioners, both as individuals and as a community, have profound impacts on the communities we serve. Furthermore, without radically altering our linguistic policies in the global health sector, we limit further the possibility for medicine and research to be discussed, published and practiced in local languages. Without addressing the current hegemony of major languages, in particular English, in the global health sphere, we risk perpetuating health inequities in every corner of the globe. We urge the promotion of a new model of global health where multilingualism is not only encouraged, but the norm.

## Data Availability

No datasets were generated or analysed during the current study.

## Abbreviations

LMICs: Low–and middle–income countries
UK: United Kingdom
USA: United States of America
AI: Artificial intelligence
